# Comparative plastome genomics and phylogenetic relationships of the genus *Trollius*

**DOI:** 10.3389/fpls.2023.1293091

**Published:** 2023-11-17

**Authors:** Jiaxin Li, Yan Du, Lei Xie, Xiaohua Jin, Zhirong Zhang, Meiqing Yang

**Affiliations:** ^1^ School of Pharmacy, Baotou Medical College, Baotou, Inner Mongolia, China; ^2^ School of Ecology and Nature Conservation, Beijing Forestry University, Beijing, China; ^3^ State Key Laboratory of Systematic and Evolutionary Botany, Institute of Botany, Chinese Academy of Sciences, Beijing, China; ^4^ Germplasm Bank of Wild Species, Kunming Institute of Botany, Chinese Academy of Sciences, Kunming, Yunnan, China

**Keywords:** *Trollius*, plastome, comparative analysis, divergent hotspots, phylogenetic relationships

## Abstract

*Trollius*, a genus in the Ranunculaceae family, has significant medicinal and ornamental value. It is widely distributed in China with 16 different species accepted. However, due to the lack of enough samples and information sites, the molecular phylogenetic relationships of *Trollius* have been unresolved till now. Here we sequenced, assembled and annotated the plastomes of 16 *Trollius* species to investigate their genomic characteristics, inverted repeat (IR) boundaries, sequence repeats, and hypervariable loci. In addition, the phylogenetic relationships of this genus was reconstructed based on the whole plastomes and the protein-coding sequences data-sets. The plastomes of *Trollius* ranged between 159,597 bp and 160,202 bp in length, and contained 113 unique genes, including 79 protein coding, 30 tRNA, and 4 rRNA. The IR boundaries were relatively conserved within the genus *Trollius*. 959 simple sequence repeats and 657 long sequence repeats were detected in the *Trollius* plastomes. We identified 12 highly polymorphic loci (Pi > 0.0115) that can be used as plastid markers in molecular identification and phylogenetic investigation of the genus. Besides, *Trollius* was a monophyletic group with the earliest divergence clade being *Trollius lilacinus* Bunge, and the remaining species were divided into two strongly-supported clades. The phylogeny in our study supported the traditional classification systems based on the color of sepal, but not the previous classification system based on the types and relative lengths of the nectaries, and distribution. The genomic resources provided in our study can be used in the taxonomy of the genus *Trollius*, promoting the development and utilization of this genus.

## 1 Introduction

*Trollius* L. are perennial herbs of the Ranunculaceae family, with more than 30 identified species distributed mainly in the temperate and cold temperate zones of the Northern Hemisphere (Doroszewska, 1974). East Asia is considered the center of distribution for this genus (Heo and Suh, 2008; Kadota, 2016), with 16 species found in alpine mountains of northeast, north, northwest, and southwest China (Ding et al., 2003), eight of which are endemic. The species *Trollius chinensis* Bunge, *T. macropetalus* Fr., *T. ledebourii* Reichenbach, and others in the genus have high medicinal value in traditional Chinese and Mongolian medicine (Yu, 2016). They were reported as effective remedies for oral issues, throat swelling, earache, and eye brightening in ancient China (He et al., 2023). In modern pharmacological investigations, chemical components found in *Trollius* are mostly anti-inflammatory, anti-viral, antioxidant, anti-tumor, and hypoglycemic (Witkowska-Banaszczak, 2015; Feng et al., 2022a), which offer promising applications in pharmacology. In addition, *Trollius* plants are commonly used for ornamental purposes due to their aesthetic appeal in landscaping and gardening (Zhang et al., 2016).

The genus *Trollius* was characterized by high morphological variability and frequent natural hybridization, which posed challenges to the classification based on traditional methods such as morphology and geographic distribution (Li, 1995). Prantl (1891) grouped 16 species of *Trollius* distributed in China into two sections based on the color of the sepal, sect. *Hegemone* (light purple or light blue sepals) and sect. *Trollius* (yellow sepals). Of these, the sect. *Hegemone* contained only one species *T. lilacinus*, and the sect. *Hegemone* included the rest 15 species. This treatment was adopted in Flora Republicae Popularis Sinicae by Wang (1979). *Trollius* was divided into seven sections (*Pumilotrollius*, *Acaulitrollius*, *Yunnanotrollius*, *Longipetala*, *Trollius*, *Insulaetrollius* and *Laxotrollius*) based on the types and relative lengths of the nectaries, distribution, and some other floral characters (Doroszewska, 1974). Three pollen types within *Trollius* were suggested by the palynological results (Lee and Blackmore, 1992). However, most characters were proved to be homoplasious and variable within species (Pellmyr, 1992). The subsequent researchers attempted to use molecular markers to address these issues. Després et al. (2003) used multiple markers, including nuclear DNA sequences (ITS1, 5.8S and ITS2) and plastid DNA sequences (*trnL* intron and *trnL-F*) as well as AFLP, to resolve the phylogenetic relationships of *Trollius*; Wang et al. (2010) combined plastid DNA markers (*matK* and *trnL*-*F*), nuclear DNA marker (ITS) and 17 morphological characters to investigate the phylogenetic relationships of *Trollius*. However, due to insufficient information of those markers, the phylogenetic relationships within the genus have not been well resolved yet.

Plastomes are multifunctional organelles in terrestrial plants, algae and a few protozoa, and a place for photosynthesis, which carry out photosynthesis as well as perform functions such as the synthesis of proteins, starches, and pigments (Zheng et al., 2020). In angiosperm plants, plastomes are generally 107^_^218 kb in size, encoding about 110^_^130 genes in a closed-loop tetrameric structure: two inverted repeats (IRa and IRb), one large single-copy (LSC) region and one small single-copy (SSC) region (Kolodner and Tewari, 1979; Asaf et al., 2017). Compared with the nuclear genome, the plastomes are inherited from one parent (maternal inheritance in most angiosperms) and have evolved more slowly. Taken together, the plastomic sequence has obvious advantages such as conserved composition and structure, relatively small genome for easy sequencing, uniparental inheritance, and moderate base variation rate. With the rapid development of high-throughput technology, plastomic data was widely used as a potentially useful tool in studies of population genetics, phylogeography, phylogeny, and species identification (Daniell et al., 2016; Xiong et al., 2021; Yang et al., 2023). A series of studies have shown that the plastomic data is effective in addressing the phylogenetic relationships among Ranunculaceae members at different taxonomic levels (Kong et al., 2017; Liu et al., 2018; He et al., 2019; Zhai et al., 2019; Li et al., 2020). In this study, we sequenced the plastomes from 16 species of *Trollius*, conducted comparative genomics and phylogenomic analyses based on these data. The main objectives of this study were: (1) to study the plastome characteristics of the genus *Trollius*; (2) to investigate variations of simple sequence repeats (SSRs) and long sequence repeats (LSRs) throughout the genus; (3) to screen highly variable regions from the plastomes as candidate DNA barcodes for the identification of species in the genus *Trollius*; (4) to construct a phylogenetic tree to elucidate the phylogenetic relationships of the genus *Trollius*.

## 2 Materials and methods

### 2.1 Sample collection, DNA extraction and sequencing

The materials of 16 *Trollius* species were obtained from the wild, herbarium of PE (Herbarium, Institute of Botany, CAS) and KUN (Herbarium, Kunming Institute of Botany, CAS), which covers six (*Pumilotrollius*, *Acaulitrollius*, *Yunnanotrollius*, *Longipetala*, *Trollius* and *Insulaetrollius*) of the seven sections in Doroszewska’s (1974) classification system. A list of the sources of plastome sequences was provided in **Table 1**. Total DNA extraction was performed using the Plant DNA Extraction Kit (TIANGEN). The quality of DNA was assessed by 1.0% agarose gel electrophoresis, and the concentration was measured using the Qubit 3.0 fluorescence quantifier (Thermo Fisher Scientific, USA). The total genomic DNA was assayed, and an insert library of approximately 350 bp in length was constructed. Paired-end sequencing with a read length of 150 bp was performed using the DNBSEQ-T7 platform. The raw reads underwent quality control using the NGS QC ToolKit (Patel and Jain, 2012) with default parameters. Adapters and low-quality reads were filtered out to obtain clean reads for further analysis.

**Table 1:** Information for sampled sequences in this study.

Species	locality	Voucher	Genbank accession	Reference
*Trollius lilacinus*	Xinjiang	17CS16246	OR449287	In this study
*Trollius pumilus*	Gansu	01957329	OR449290	In this study
*Trollius altaicus*	Xinjiang	00466745	OR449279	In this study
*Trollius asiaticus*	Xinjiang	00466753	OR449280	In this study
*Trollius buddae*	Gansu	00466760	OR449281	In this study
*Trollius dschungaricus*	Xinjiang	01957240	OR449283	In this study
*Trollius japonicus*	Jilin	ZhouHC1386	OR449285	In this study
*Trollius macropetalus*	Jilin	01596585	OR449288	In this study
*Trollius micranthus*	Yunnan	01659232	OR449289	In this study
*Trollius vaginatus*	China	00467277	OR449293	In this study
*Trollius ledebourii*	Inner Mongolia	01957278	OR449286	In this study
*Trollius yunnanensis*	Yunnan	01659229	OR449294	In this study
*Trollius chinensis*	Hebei	02031193	OR449282	In this study
*Trollius taihasenzanensis*	Taiwan	YOU546	OR449292	In this study
*Trollius ranunculoides*	Sichuan	01900525	OR449291	In this study
*Trollius farreri*	Shaanxi	TianXH427	OR449284	In this study
*Calathodes oxycarpa*	Hubei	ZW12-004	NC041475	(Zhai et al., 2019)
*Adonis amurensis*	Jilin	sfxyyyxycjzh20200429	NC056353	(Zhang et al., 2021)
*Adonis sutchuenensis*	Shaanxi	SL12-053	NC041474	(Zhai et al., 2019)
*Adonis coerulea*	Sichuan	H. J. Liu I-1109	MK253469	(He et al., 2019)

### 2.2 Plastome assembly and annotation

The plastomes were assembled using the GetOrganelle v. 1.7.5 (Jin et al., 2020) and were annotated by CPGAVAS2 (Shi et al., 2019) with *T. chinensis* (NC_031849) as a reference. The annotation errors were manually corrected according to plastomes of congeneric species in Geneious v. 9.0.2 (Kearse et al., 2012). The assembled and annotated sequences of the whole plastomes of 16 *Trollius* species were submitted to the GenBank database (**Table 1**). Finally, the maps of annotated plastomes were drawn using the online program OGDRAW v. 1.3.1 (Greiner et al., 2019).

### 2.3 Comparative genomic analysis

Analysis of the boundaries of plastomes was conducted using the IRscope tool (Amiryousefi et al., 2018) to visualize the inverted repeats. The reference genome used for this analysis was the plastome of *T. chinensis*. Pairwise alignments were performed using the online tool mVISTA (Frazer et al., 2004) in Shuffle-LAGAN mode. Subsequently, the aligned plastome sequences were analyzed to compute the nucleotide diversity (Pi) using DnaSP v. 6.12.03 (Rozas et al., 2017). A window length of 600 bp with a step size of 200 bp was employed, and a plot was generated based on the computed data.

### 2.4 Sequence repeats analysis

Simple sequence repeats (SSRs) in the 16 plastome sequences of *Trollius* were examined using the Perl script in MISA (Beier et al., 2017). Minimum thresholds were set for different types of SSRs: mononucleotide, dinucleotide and trinucleotide SSRs required at least ten, five and four repeat units respectively, and tetra-, penta-, and hexa-nucleotide SSRs all required at least three repeat units. The detection of long sequence repeats (LSRs), including forward (F), reverse (R), palindromic (P) and complementary repeats (C), was performed using the online software REPuter (Kurtz et al., 2001). The settings were configured to identify repeat sizes greater than 30 bp with a Hamming distance of 3.

### 2.5 Phylogenetic analysis

*Calathodes oxycarpa* Sprague, *Adonis amurensis* Regel et Radde, *A. sutchuenensis* Franch., and *A. coerulea* Maxim. were selected as outgroups according to the phylogenetic framework provided by Zhai et al. (2019). Phylogenetic trees were inferred using the maximum parsimony (MP), maximum likelihood (ML), and Bayesian inference (BI) based on the following three data sets: (1) the whole plastome sequences, (2) the extracted sequences representing all protein-coding sequences (CDS), (3) the nuclear ribosomal DNA (nrDNA). To find more promising DNA markers, we also constructed the phylogenetic tree using maximum likelihood (ML) method based on the 11 individual matrices representing 11 highly polymorphic loci identified. Another highly polymorphic locus *ycf1-ndhF* was excluded because some sites in this region are ambiguously aligned. Sequence alignment was performed with MAFFT v. 7.40 (Katoh and Standley, 2013). Phylogenetic relationships reconstruction analysis was conducted using RAxML (Stamatakis, 2014) with ML, employing the GTR+G nucleotide substitution model and 1000 bootstrap replicates. MP analysis was performed using PAUP 4.0b10 (Wilgenbusch and Swofford, 2003) with a heuristic search algorithm using TBR branch exchange, with 100 repetitions. The analysis produced 50% majority-rule consensus trees and strict consensus trees, and bootstrap analysis was performed with 1000 replicates. Bayesian inference (BI) analysis was carried out using MrBayes v. 3.2 (Ronquist et al., 2012) software. The best model and parameters were determined using Modeltest v. 3.7 (Posada and Buckley, 2004). Four MCMC chains were run simultaneously for 10,000,000 generations, with tree sampling every 1000 generations. After discarding the first 25% samples as burn-in, posterior probabilities (PP) were calculated for a consensus tree. The average standard deviation of split frequencies between the two runs was 0.008, and ESS values were above 200 for all individual MCMC runs.

## 3 Results

### 3.1 Plastome features of *Trollius*

**Figure 1:**
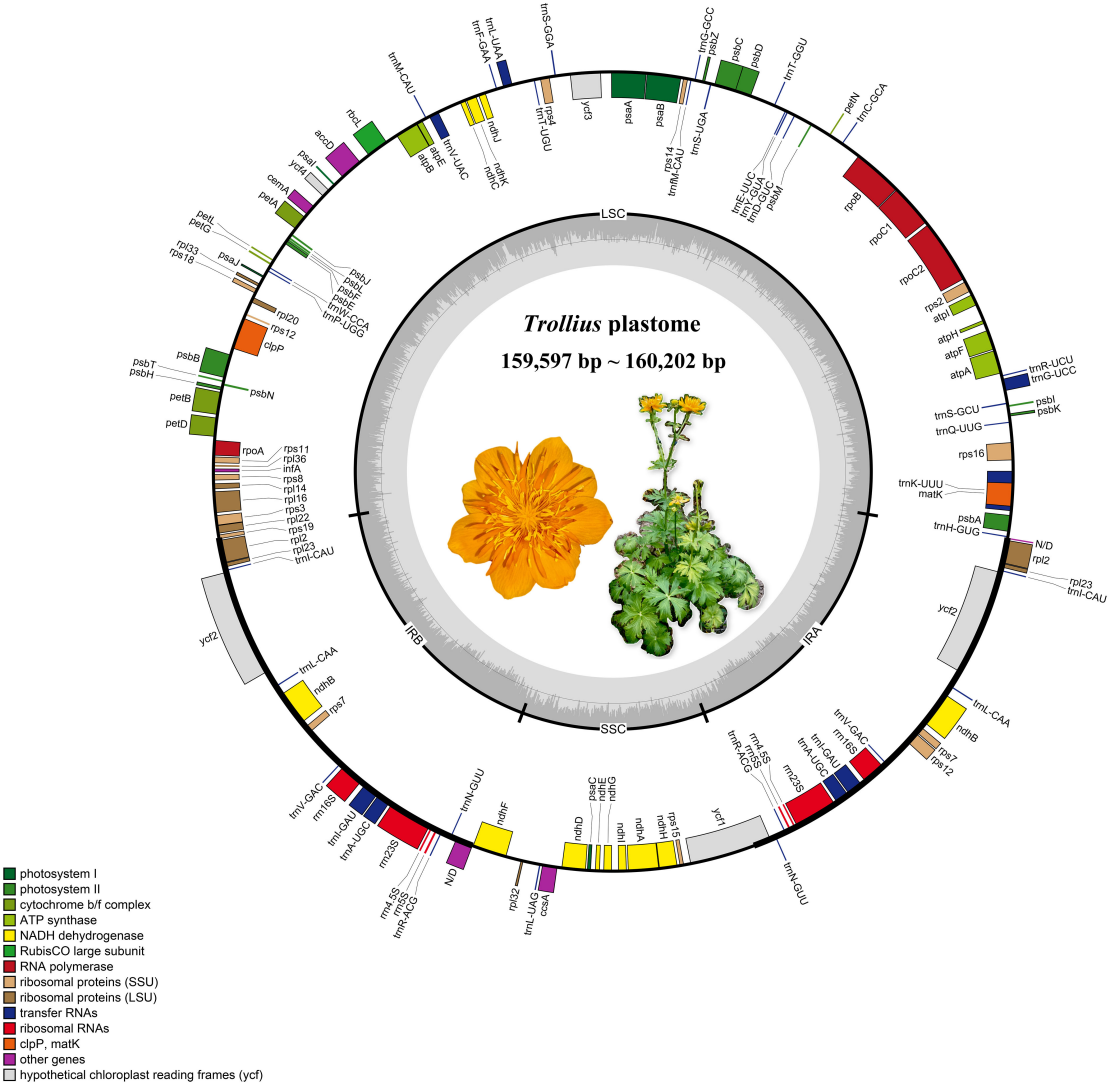
The plastomes gene map of *Trollius*. The genes transcribed clockwise and counterclockwise are on the inner and outer side the circle of the map, respectively. Gene functions labeled on the legend and the gray dashed line in the inner circle reflects the GC content. It is a color image of *Trollius chinensis* in the circle, a representative species of the genus.

The length of the plastomes varied slightly among the 16 species that were analyzed, ranging from 159,597 bp of *T. micranthus* Hand.-Mazz. to 160,202 bp of *T. lilacinus* Bunge (**Figure 1**; **Table 2**). The 16 *Trollius* plastomes were all four-part in structure: a pair of inverted repeat regions (IRa and IRb) of 26,490^_^26,610 bp separated by one large single copy (LSC) region of 88,091^_^88,712 bp and one small single copy (SSC) region of 18,083^_^18,497 bp. The GC content in the whole plastomes of *Trollius* ranged from 38.0 to 38.1%, with a uniform percentage of 43.1% in IRs, 36.2-36.3% in LSC, and 31.8^_^32.0% in SSC. These findings indicated that the plastomes of *Trollius* species have nearly identical in terms of GC content. In addition, the GC content of IR regions was higher than that of LSC region and SSC region.

**Table 2:** The basic plastome characteristics of 16 *Trollius* species.

Species	Genome (bp)	GC %	LSC (bp)	GC % (LSC)	IR (bp)	GC % (IR)	SSC (bp)	GC% (SSC)	Gene total	Protein encoding	tRNA genes	rRNA genes
*T. lilacinus*	160,202	38.0%	88,712	36.3%	26,502	43.1%	18,486	31.8%	113	79	30	4
*T. pumilus*	159,598	38.0%	88,091	36.3%	26,505	43.1%	18,497	31.8%	113	79	30	4
*T. altaicus*	159,986	38.1%	88482	36.3%	26,576	43.1%	18,352	32.0%	113	79	30	4
*T. asiaticus*	160,002	38.1%	88523	36.3%	26,526	43.1%	18,427	32.0%	113	79	30	4
*T. buddae*	159,688	38.0%	88215	36.3%	26,508	43.1%	18,457	31.8%	113	79	30	4
*T. dschungaricus*	159,816	38.1%	88,567	36.3%	26,583	43.1%	18083	32.0%	113	79	30	4
*T. japonicus*	160,061	38.0%	88,492	36.3%	26,582	43.1%	18,405	31.9%	113	79	30	4
*T. macropetalus*	159,947	38.1%	88,422	36.3%	26,571	43.1%	18,383	32.0%	113	79	30	4
*T. micranthus*	159,597	38.1%	88,239	36.3%	26,490	43.1%	18,378	31.8%	113	79	30	4
*T. vaginatus*	159,724	38.1%	88,286	36.3%	26,506	43.1%	18,426	31.8%	113	79	30	4
*T. ledebourii*	160,025	38.0%	88,488	36.3%	26,581	43.1%	18,375	32.0%	113	79	30	4
*T. yunnanensis*	159,717	38.0%	88,285	36.3%	26,506	43.1%	18,420	31.8%	113	79	30	4
*T. chinensis*	160,009	38.0%	88,487	36.3%	26610	43.1%	18,302	32.0%	113	79	30	4
*T. taihasenzanensis*	160019	38.0%	88,564	36.2%	26,540	43.1%	18,375	32.0%	113	79	30	4
*T. ranunculoides*	159,616	38.0%	88,116	36.3%	26,505	43.1%	18,490	31.8%	113	79	30	4
*T. farreri*	159,629	38.0%	88,178	36.3%	26,503	43.1%	18,445	31.8%	113	79	30	4

There were 113 genes in the plastomes of *Trollius*, including 79 protein-encoding genes, 30 transfer RNA genes and 4 ribosomal RNA genes (**Tables 2**, **3**). The following genes have been duplicated in the IR regions: six protein-coding genes (*ndhB*, *rpl23*, *rps7*, *rps12*, *ycf2* and *rpl2*), seven tRNA genes (*trnI-CAU*, *trnL-CAA*, *trnV-GAC*, *trnI-GAU*, *trnA-UGC*, *trnR-ACG* and *trnN-GUU*) and all four rRNA genes. 15 genes (*atpF*, *ndhA*, *ndhB*, *petB*, *petD*, *rpl2*, *rpl16*, *rpoC1*, *rps12*, *rps16*, *trnA-UGC*, *trnG-UCC*, *trnI-GAU*, *trnK-UUU*, *trnV-UAC*) contained one intron, and two genes (*clpP* and *ycf3*) contained two introns.

**Table 3:** Genes of *Trollius* plastome.

Function of genes	Group of genes	Name of genes
Photosynthesis	ATP synthase	*atpA*, *atpB* ^(1)^, *atpE*, *atpF* ^(1)^, *atpH*, *atpI*
	NADH dehydrogenase	*ndhA* ^(1)^, *ndhB* *, *ndhC*, *ndhD*, *ndhE*, *ndhF*, *ndhG*, *ndhH*, *ndhI*, *ndhJ*, *ndhK*
	cytochrome c synthesis	*ccsA*
	Assembly/stability of photosystem I	*ycf3* ^(2)^, *ycf4*
	photosystem I	*psaA*, *psaB*, *psaC*, *psaI*, *psaJ*
	photosystem II	*psbA*, *psbB*, *psbC*, *psbD*, *psbE*, *psbF*, *psbH*, *psbI*, *psbJ*, *psbK*, *psbL*, *psbM*, *psbN*, *psbT*, *psbZ*
	cytochrome b/f complex	*petA*, *petB* ^(1)^, *petD* ^(1)^, *petG*, *petL*, *petN*
	Rubisco	*rbcL*
Self-replication	ribosomal proteins	*rps2*, *rps3*, *rps4*, *rps7* *, *rps8*, *rps11*, *rps12* * ^(1)^, *rps14*, *rps15*, *rps16* ^(1)^, *rps18*, *rps19*, *rpl2* *^(1)^, *rpl14*, *rpl16* ^(1)^, *rpl20*, *rpl22*, *rpl23* *, *rpl32*, *rpl33*, *rpl36*
	transcription	*rpoA*, *rpoB*, *rpoC1* ^(1)^, *rpoC2*
	translation initiation	*infA*
	Ribosomal RNAs	*rrn5* *, *rrn4.5* *, *rrn16* *, *rrn23* *
	Transfer RNAs	*trnA-UGC* *^(1)^, *trnC-GCA*, *trnD-GUC*, *trnE-UU*, *trnF-GAA*, *trnfM-CAU*, *trnG-GCC*, *trnG-UCC* ^(1)^, *trnH-GUG*, *trnI-CAU* *, *trnI-GAU* * ^(1)^, *trnK-UUU* ^(1)^, *trnL-CAA* *, *trnL-UAA*, *trnL-UAG*, *trnM-CAU*, *trnN-GUU* *, *trnP-UGG*, *trnQ-UUG*, *trnR-ACG* *, *trnR-UCU*, *trnS-GCU*, *trnS-GGA*, *trnS-UGA*, *trnT-GGU*, *trnT-UGU*, *trnV-GAC* *, *trnV-UAC* ^(1)^, *trnW-CCA*, *trnY-GUA*
Other genes	RNA processing	*matK*
	carbon metabolism	*cemA*
	Fatty acid synthesis	*accD*
	proteolysis	*clpP* ^(2)^
Unknown function	conserved reading frames	*ycf1*, *ycf2* *

^(1)^ Gene with one intron;

^(2)^ Gene with two introns;

^*^ Number of copies of multi-copy genes.

### 3.2 Inverted repeat expansion and contraction

**Figure 2:**
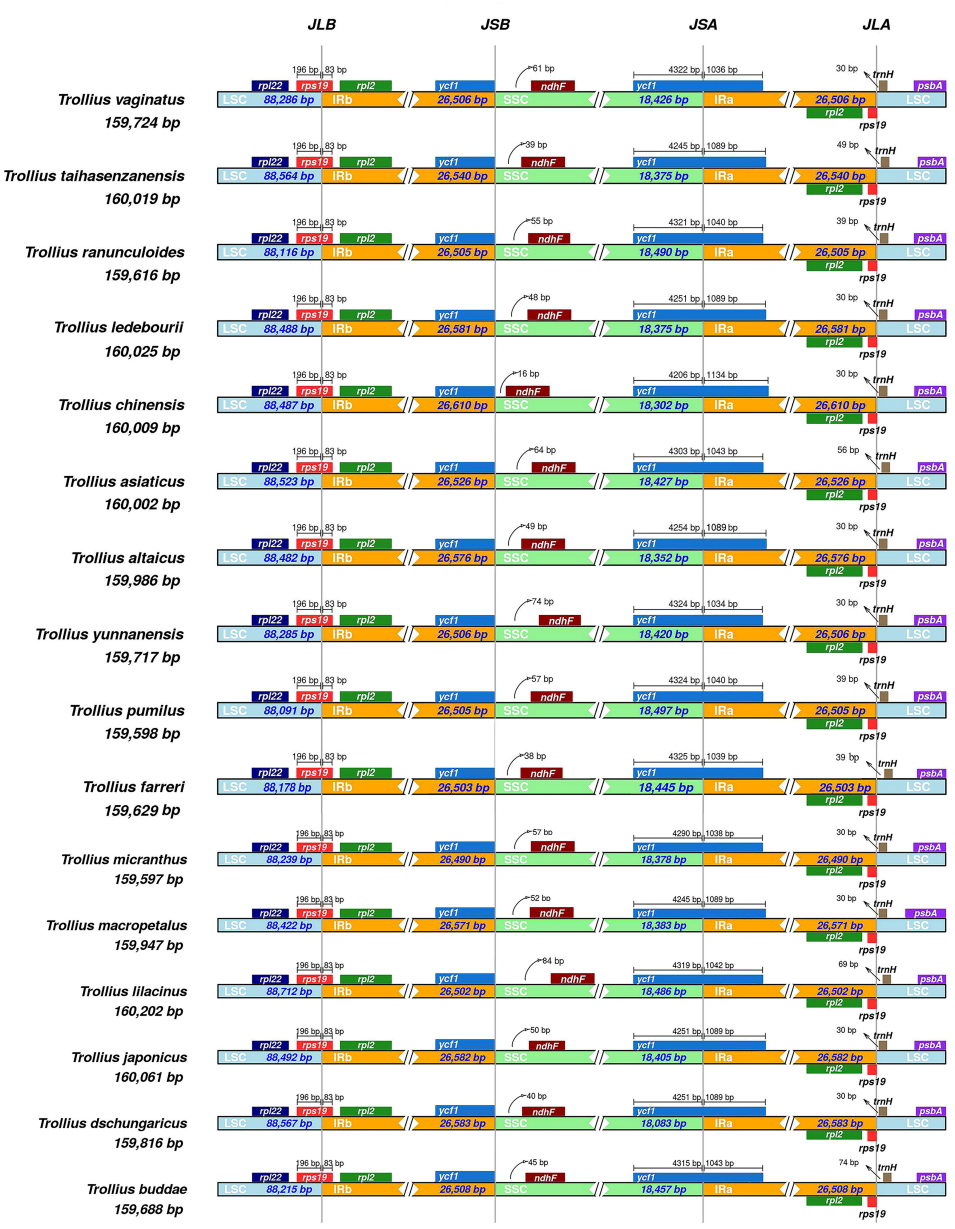
Comparison of the borders of LSC, SSC, and IR regions of plastomes in 16 *Trollius* species.

The comparative results of contraction and expansion of IR/SC boundaries within 16 species of *Trollius* were shown in **Figure 2**. We discovered that the four boundaries of the plastome were relatively conserved. The LSC/IRb (JLB) boundary was found to be located within the *rps19* gene for all species, and the gene size was highly consistent with 196 bp in the LSC region and 83 bp expansion into the IRb region. The distances between the LSC/IRa (JLA) boundary and *trnH* were 30^_^74 bp, 9 species (*T. altaicus* C. A. Mey., *T. dschungaricus* Regel, *T. japonicus* Miq., *T. macropetalus*, *T. micranthus*, *T. vaginatus* Hand.-Mazz., *T. ledebourii*, *T. yunnanensis* (Franch.) Ulbr., and *T. chinensis*) were all located 30 bp away from the JLA boundary. At the SSC/IRa (JSA) boundary, the *ycf1* gene was observed to span the SSC region into the IRa region. The gene length in the SSC region ranged from 4206 to 4325 bp with expansion of 1034^_^1134 bp into the IRa region. Specifically, the *ycf1* genes of *T. japonicus*, *T. dschungaricus*, and *T. ledebourii* all showed the same characteristics at the JSA boundary with a length of 4251 bp located in the LSC region and 1089 bp in the IRa region. *Trollius taihasenzanensis* Masamune and *T. macropetalus* had the same JSA boundary with a length of 4245 bp in the SSC region and 1089 bp in the IRa region. The distance between the IRb/SSC (JSB) boundaries and *ndhF* varied from 16 to 84 bp. Only *T. pumilus* D. Don and *T. micranthus* were 57 bp from the JSB boundaries.

### 3.3 Characteristics of sequence repeats

**Figure 3:**
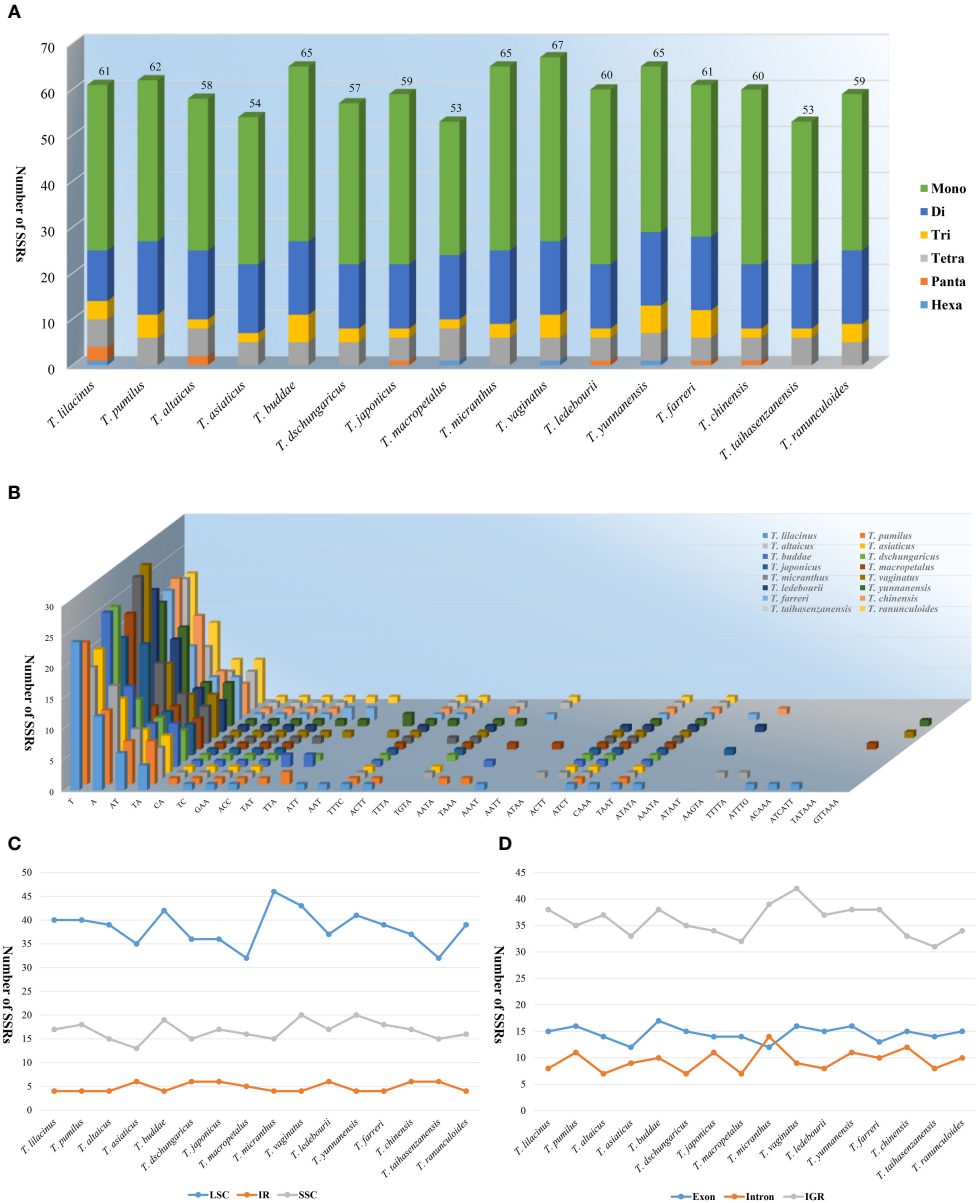
Comparison of the types and distribution of SSRs in the 16 *Trollius* plastomes. **(A)** Number of SSR repeat types. **(B)** Number of identified SSR motifs in different repeat class types. **(C)** Number of SSRs in the LSC, IR, SSC. **(D)** Number of SSRs in the intergenic regions (IGRs), exons and introns.

In the SSR analysis, the number of SSRs was relatively consistent among the 16 species of the genus *Trollius*. *Trollius taihasenzanensis* and *T. macropetalus* exhibited the lowest number of SSRs (53), with *T. vaginatus* having the highest number of SSRs (67), as shown in **Figure 3**. The number of mononucleotide (29^_^40), dinucleotide (11^_^16), trinucleotide (2^_^6) and tetranucleotide (5^_^7) SSRs varied among the species. Additionally, pentanucleotide and hexanucleotide SSRs both were present in *T. lilacinus*, and pentanucleotide SSRs were detected in *T. altaicus*, *T. japonicus*, *T. ledebourii*, and *T. chinensis*. Furthermore, hexanucleotide SSRs were identified in *T. macropetalus*, *T. vaginatus*, and *T. yunnanensis* (**Figure 3A**). Generally, mononucleotides were the most abundant, exceeding half of the total (58.92%), followed by dinucleotides (24.71%) and tetranucleotides (5.84%). A/T was the predominant nucleotide composition in mononucleotide SSRs. In dinucleotide SSRs, the most frequent type was AT/TA. Additionally, the most common type of tetranucleotide SSR was TTTC (**Figure 3B**). The results of distribution of SSRs indicated that the LSC region (32^_^46) had the highest number of SSRs, followed by the SSC region (13^_^20), while the IR region (4^_^6) had the lowest number of SSRs (**Figure 3C**). Besides, the majority of SSRs were found in the intergenic regions (IGRs) (31^_^42), with fewer SSRs detected in the exon (12^_^17) and intron (7^_^14) regions. In particular, we observed that the SSRs of *T. micranthus* were more frequently located in the intron region than the exon region (**Figure 3D**).

**Figure 4:**
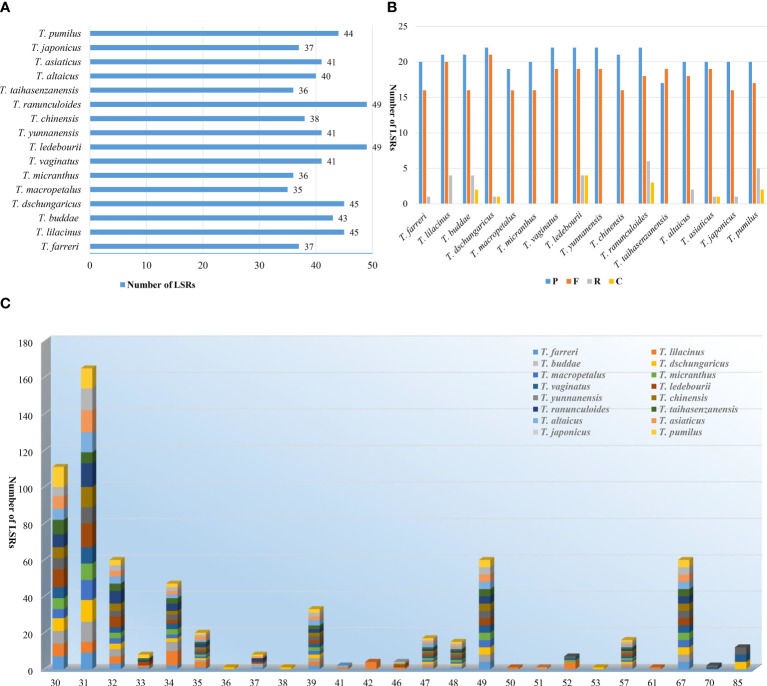
LSRs in the plastomes of 16 *Trollius*. **(A)** Number of LSRs. **(B)** Number of different LSRs types. **(C)** Number of LSRs of different lengths.

Totally 657 LSRs were detected in 16 *Trollius* species, among which *T. ledebourii* and *T. ranunculoides* Hemsl. had the highest number of 49 and *T. macropetalus* had the least number of 35 (**Figure 4A**). Palindrome and forward LSRs were observed in all 16 species examined, but reverse and complement LSRs were not present in all species. Specifically, *T. macropetalus, T. micranthus, T. vaginatus, T. yunnanensis, T. chinensis* and *T. taihasenzanensis* showed no reverse and complement LSRs (**Figure 4B**). Among the LSRs types, palindrome LSRs were the most abundant (17^_^22), followed by forward LSRs (16^_^21). LSRs lengths for 16 species ranged from 30^_^85. Length of 31 bp was the most common long sequence repeats, followed by a length of 30 bp. Remarkably, *T. lilacinus* lacked LSRs of 48, 49, and 67 bp lengths that were shared by the other 15 species (**Figure 4C**).

### 3.4 Sequence divergence and hotspots

**Figure 5:**
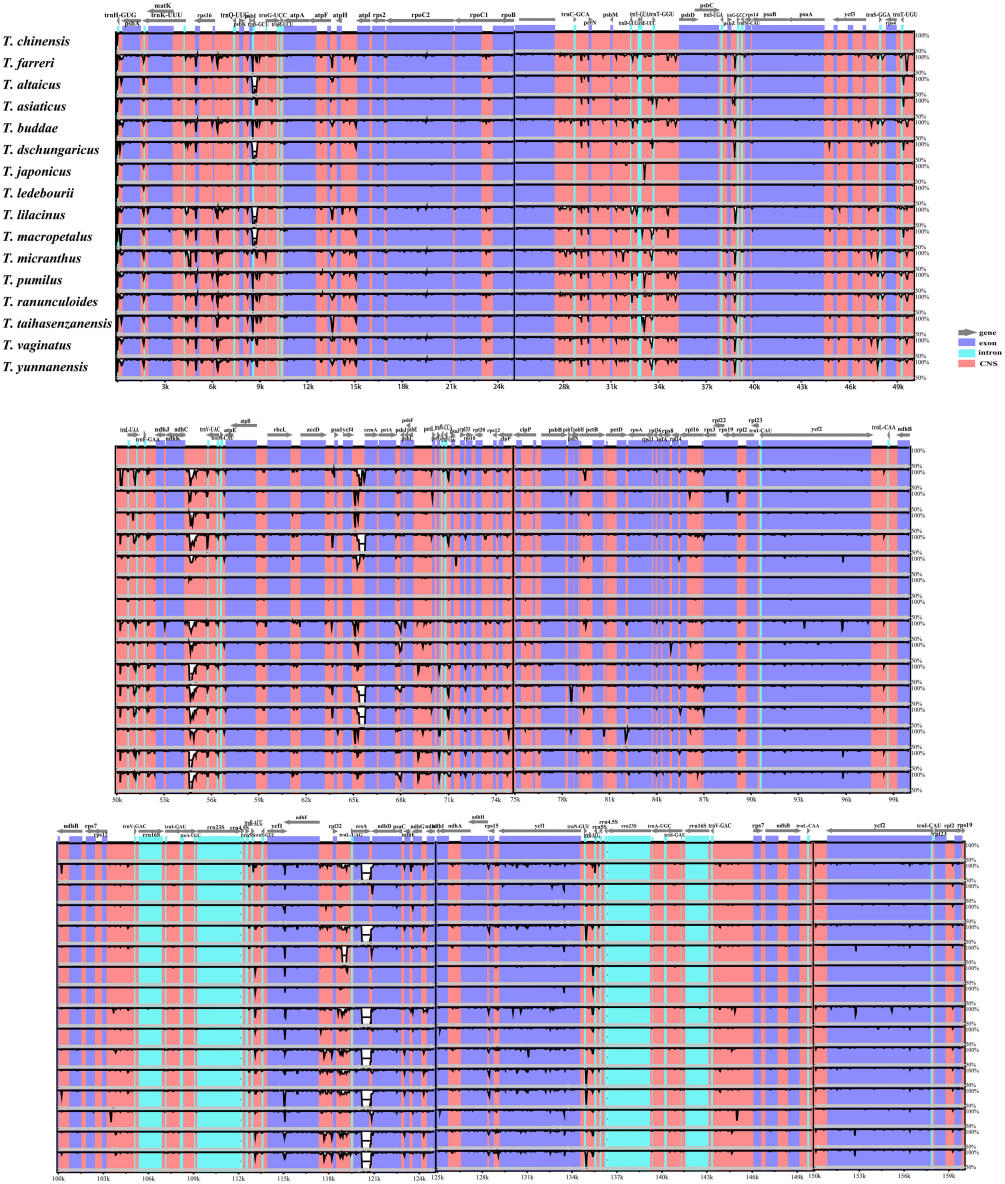
The plastomes of 16 *Trollius* species were aligned using the Shuffle-LAGAN alignment algorithm in mVISTA and *T. chinensis* was selected as a reference. The Y-axis represents the percentage of identity between 50% and 100%.

We compared the similarity of plastomes within the genus *Trollius* using the mVISTA program, with *T. chinensis* as a reference (**Figure 5**). Our analysis revealed a relatively high level of sequence conservation in genome structure and gene order across the 16 *Trollius* species, with no significant large-scale inversions or gene rearrangements. However, we observed relatively low homology in five regions: *rpl32*-*trnL*, *ycf4*-*cemA*, *ndhC*-*trnV*, *trnS*-*trnG*, and *ccsA*-*ndhD*, with partial segments displaying homology below 50%. Most of these highly variable regions were found within the conserved non-coding sequence (CNS). Moreover, we observed that the IR regions exhibited higher conservation compared to the single-copy (SC) regions.

**Figure 6:**
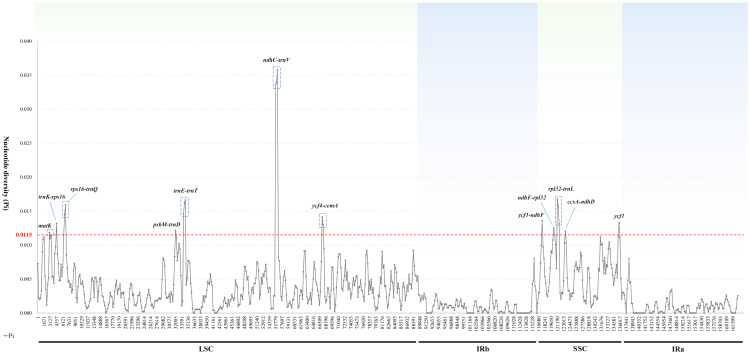
Sliding window analysis of the 16 *Trollius* plastomes. X-axis: position of the midpoint of a window; Y-axis: nucleotide diversity of each window.

To identify sequence divergence hotspots, we calculated nucleotide diversity (Pi) values by DNAsp (**Figure 6**). By setting the window size to 600 bp, we found that Pi values within the region ranged from 0 to 0.03574, with the *ndhC*-*trnV* region exhibiting the highest Pi value (0.03574). Obviously, the mean Pi values in the IR, LSC, and SSC regions were 0.000964, 0.004068, and 0.006403, respectively. The mean Pi values observed in the IR region were lower than those in the SSC and LSC regions indicated that the IR region was more conserved than SC regions. Our analysis determined that 12 highly variable regions (Pi > 0.0115) were located in the SC region, including *ndhC*-*trnV*, *rpl32*-*trnL*, *rps16*-*trnQ*, *trnE*-*trnT*, *ycf4*-*cemA*, *ycf1*-*ndhF*, *ycf1*, *trnK*-*rps16*, *ndhF*-*rpl32*, *psbM*-*trnD*, *ccsA*-*ndhD*, and *matK*. Specifically, all of these regions except for *matK* and *ycf1* were located in IGRs.

### 3.5 Phylogenetic analysis

**Figure 7:**
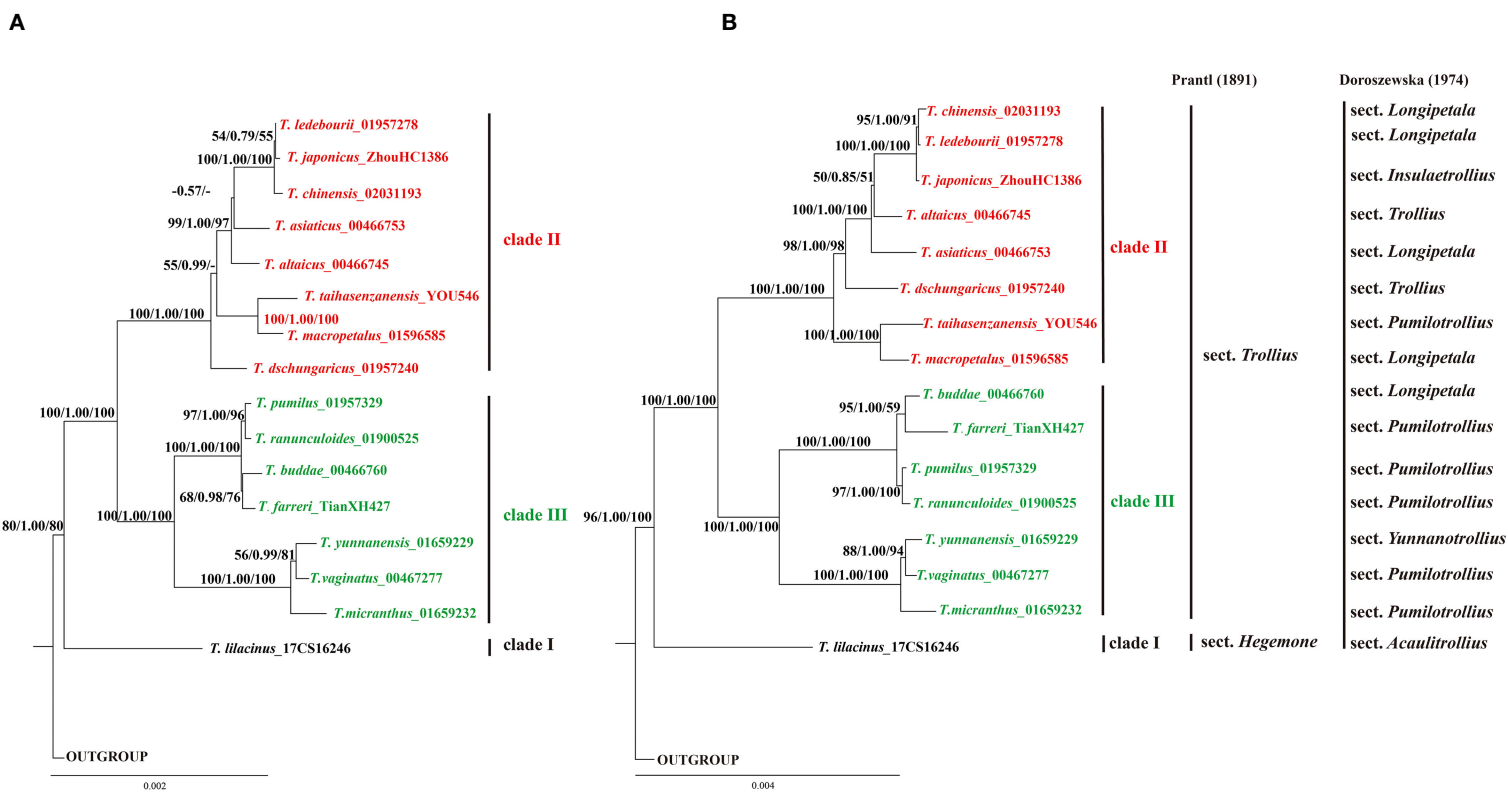
The phylogenetic tree of *Trollius* was reconstructed using maximum likelihood (ML), Bayesian inference (BI) and maximum parsimony (MP) methods. Each branch was annotated with three values: the ML bootstrap values, the Bayesian posterior probability, and the MP bootstrap values. **(A)** Phylogenetic tree constructed using the protein-coding sequences (CDS). **(B)** Phylogenetic tree constructed using the whole plastomes. Traditional classification systems of Prantl (1891) and Doroszewska (1974) were showed on the right vertical lines.

This study reconstructed phylogenetic tree of 16 *Trollius* species using maximum likelihood, Bayesian inference, and maximum parsimony methods based on three different sequence matrices. The phylogenetic tree based on the whole plastome sequences exhibited similar topologies with the phylogenetic tree based on CDS, and showed higher support values of some nodes, as shown in **Figure 7**. Due to a low level of sequence variation, the nrDNA tree was poorly resolved (**Figure S1**). The phylogenetic trees of eight highly polymorphic loci (**Figures S2A, B, D, E, F, G, J, K**) supported that the genus *Trollius* formed three clades (clade I, clade II and clade III), of which *ycf1*, *rps16*-*trnQ*, *psbM*-*trnD* gene trees (**Figures S2I, C, H**) were fully consistent with the phylogenetic tree based on the whole plastome sequences (**Figure 7**). Therefore, the results of the phylogeny in this study were mainly based on the whole plastomes. *Trollius* formed a monophyletic group with early diverging clade of *T. lilacinus* (clade I), which was strongly supported by three methods (MLBS = 100%; PP = 1.00; MPBS = 100%). The remaining 15 *Trollius* species were divided into two clades: the clade II contained most of the species from temperate zones (MLBS = 100%; PP = 1.00; MPBS = 100%), including *T. dschungaricus*, *T. asiaticus* L., *T. chinensis*, *T. ledebourii*, *T. japonicus*, *T. altaicus*, and *T. macropetalus*. The clade III included most of the species from subtropical zones (MLBS = 100%; PP = 1.00; MPBS = 100%), such as *T. farreri* Stapf, *T. pumilus*, *T. ranunculoides*, *T. micranthus*, *T. yunnanensis* and *T. vaginatus*.

## 4 Discussion

### 4.1 Characteristics of plastome in *Trollius*

The plastomes of the 16 *Trollius* species sequenced in our study showed a typical quadripartite structure, consistent with other species in the family Ranunculaceae (Zhai et al., 2019). These genomes were larger than the average plastome size of land plants (151 kb), ranging from 159,597 to 160,202 bp, and their GC content (38.0^_^38.1%) was higher than the average GC content (36.3%) of land plant (Guo et al., 2021). In addition, we observed a significant variation in GC content in different regions of the plastomes, with the highest GC content observed in IR regions. This difference in GC content may be attributed in part to the fact that the four rRNA genes, which have a lower proportion of AT nucleotides, are all located in the IR regions of the plastomes (Palmer and Thompson, 1981; Wang et al., 2008; Yang et al., 2014).

The gene content, gene order and gene number observed in *Trollius* were consistent with those in the plastomes of most angiosperm, as reported in previous studies (Wicke et al., 2011; Daniell et al., 2016; Yao et al., 2023). We found that 16 species of *Trollius* all harbored 113 intact plastid genes, representing the largest number of plastid genes in the Ranunculaceae. We observed that the *rpl32* was present in the plastomes of *Trollius*, but absent in many distant-related species within the family such as *Adonis sutchuenensis* and *Leptopyrum fumarioides* (L.) Reichb., which indicated that *rpl32* have lost independently (Zhai et al., 2019).

Previous studies have shown that the expansion and contraction of the IR region were major contributors to changes in plastome size (Cheng et al., 2017; Li et al., 2021; Liu et al., 2022). However, we observed very similar patterns of SC/IR boundaries in *Trollius* without a clear signal of expansion or contraction in our study. Repeat sequences play an important role in plastome size in recent study (Feng et al., 2022b), yet we found that no significant differences in terms of the numbers of SSRs and LSRs (**Figures 2**, **3**). Interestingly, the IR region lengths largely supported the phylogenetic relationships. The clade II consisted of eight species with slightly longer IR region ranged from 26,526 bp to 26,610 bp (*T. dschungaricus*, *T. asiaticus*, *T. chinensis*, *T. ledebourii*, *T. japonicus*, *T. altaicus*, *T. taihasenzanensis*, and *T. macropetalus*, **Figures 2**, **7**), the clade III consisted of seven species with slightly shorter IR region ranged from 26,490 bp to 26,508 bp (*T. buddae Schipcz*., *T. farreri*, *T. pumilus*, *T. ranunculoides*, *T. micranthus*, *T. yunnanensis* and *T. vaginatus*, **Figures 2**, **7**). We speculated that these species experienced different evolutionary pressures after diverging from *T. lilacinus*, resulting in diversification evolution of the length of IR region (Zhang et al., 2013).

### 4.2 Sequence repeats analysis

SSRs in angiosperm plastomes can be highly variable at the interspecific level due to sliding strand mismatches and unconventional recombination during DNA replication on individual DNA strands (Gandhi et al., 2010; Yuan et al., 2017). Moreover, their simple and relatively conservative structure makes them frequently used as molecular markers in population genetics, polymorphism studies and species identification (Murat et al., 2011). In our study, we detected the mononucleotide SSRs accounted for the largest number of all SSRs (58.92%), and A/T repeats, AT/AT repeats, TA/TA repeats, TAAT/TAAT repeats were common in all species (**Figure 3B**). This phenomenon was also reported in other genera of the Ranunculaceae family, such as *Anemoclema* (Franch.) W.T. Wang and *Pulsatilla* Adans. (Liu et al., 2018; Li et al., 2020), which can be attributed to the easier separation of AT base pairs compared to GC base pairs during plasmid replication (Zhao et al., 2020). Additionally, we noticed that the IR region had fewer SSRs than the other regions (**Figure 3C**). This was because the repetitive nature of the IR region encouraged copy correction activity, which lowered the frequency of SSRs in this region compared to the LSC and SSC regions.

Previous studies have shown that long sequence repeats can be provide important insight into the evolutionary relationships of species (Milligan et al., 1989; Liao et al., 2021). In our study, *T. lilacinus* exhibited a unique feature: it lacked the 48, 49, and 67 bp long sequence repeats that were shared by the other species. This finding might have suggested that *T. lilacinus* was more distantly related to the other species, which can be further supported by the observed morphological characters and habitats, such as unique light purple or light blue sepals, thick and coiled fibrous root, and altitude of 2600^_^3500 in the Tianshan Mountains of Xinjiang.

### 4.3 Identification of molecular markers

Mutations in the plastome tend to occur more frequently in certain regions known as “hot spots”, and these regions can exhibit high levels of genetic variation. As a result, hot spots can be employed as specific DNA barcodes for rapid and accurate species identification (Dong et al., 2012). Generally speaking, the IR regions exhibited the lowest average sequence variation among the four regions in the plastomes of *Trollius*. The highly conserved IR regions may be associated with a higher GC content (Chen et al., 2021). Non-coding regions have more highly variable loci and high Pi values than coding regions, consistent with findings observed in other taxonomic groups (Fan et al., 2018; Sun et al., 2020). Specifically, 12 highly variable hotspots were identified, including ten intergenic spacer regions (*ndhC-trnV, rpl32-trnL, rps16-trnQ, trnE-trnT, ycf4-cemA, ycf1-ndhF*, *trnK-rps16, ndhF-rpl32, psbM-trnD, ccsA-ndhD*) and two protein-coding regions (*ycf1* and *matK*). The non-coding region of *ndhC*-*trnV* showed the highest variability (**Figure 6**), which also have been selected as highly variable regions in *Pulsatilla* and *Aconitum* L. of Ranunculaceae (Park et al., 2017; Li et al., 2020). The protein-coding region *matK* was most commonly employed in plant phylogenetic studies and identification (Li et al., 2019). The other protein-coding region *ycf1* has been reported that the most variable regions of the plastome (Dong et al., 2012), which had remarkably high nucleotide diversity in *Thalictrum* L. of Ranunculaceae (Xiang et al., 2022). The phylogenetic analysis of 11 highly variable loci indicated that *rps16*-*trnQ*, *psbM*-*trnD* and *ycf1* have great potential as molecular marker or DNA barcode for this genus.

### 4.4 Phylogenetic analyses

Due to the limited information provided by morphological characters and molecular markers, the phylogenetic relationships of *Trollius* have long been poorly resolved (Li, 1995; Després et al., 2003; Wang et al., 2010). However, we have not only effectively resolved phylogenetic relationships but also greatly improved the support values for clades of the phylogenetic tree of *Trollius* based on plastomes. Our findings strongly supported that the monophyly of *Trollius* composed of three clades. *Trollius lilacinus* was the earliest divergent clade (clade I) within the genus, which was supported by its unique morphological traits and distribution area, such as pollen type (Lee and Blackmore, 1992), lilac-blue or pale blue sepals, the flowers appeared before the leaves emerge, thick and coiled fibrous root, and altitude of 2600^_^3500 in the Tianshan Mountains of Xinjian (Wang, 1979).

The remaining 15 *Trollius* species were divided into two clades, clade II mainly consisting of species from temperate zones, and the clade III mostly composed of species from subtropical zones, which was congruent with the result of Wang et al. (2010). In the clade II, *T. chinensis* was closely related to *T. ledebourii* and *T. japonicus*, which shared similar morphological features, such as the flowers being single terminal or in 2^_^3 flowered umbels. Among them, *T. chinensis* and *T. ledebourii* formed a sister clade, which shared many of the same chemical components, including flavonoids like orientin and organic acids like palmitic acid (Hao, 2018). Furthermore, they were both distributed at relatively low altitudes. In the clade III, *T. micranthus*, *T. yunnanensis*, and *T. vaginatus* were more closely related to each other compared to other species, and they all had pentagonal leaves with long petioles. Among them, *T. yunnanensis* and *T. vaginatus* formed a sister clade, both of which occurred in Yunnan and Sichuan provinces of China. The sister group of *T. ranunculoides* and *T. pumilus* had overlapping ranges in Tibet (Wang, 1979). Although most species were assigned to their respective clades, the phylogenetic placement of *T. buddae* and *T. taihasenzanensis* were incongruent with their distribution areas. *Trollius buddae* was distributed in temperate zones but included in the clade III, and *T. taihasenzanensis* was distributed in subtropical zones but not included in the clade III. This phenomenon may be caused by natural hybridization reported to occur naturally in *Trollius* (Li et al., 2016). In the future, there is a critical need to integrate more sampling and nuclear genes to better understand the phylogenetic relationships.

The plastome phylogeny supported the traditional classification system of Prantl (1891) but not Doroszewska’s (1974) classification system. Prantl (1891) grouped 16 species of *Trollius* distributed in China into two sections based on the color of the sepal, sect. *Hegemone* (light purple or light blue sepals) and sect. *Trollius* (yellow sepals), which was supported by subsequent palynotaxonomic study (Lee and Blackmore, 1992). Pollen grains of Sect. *Hegemone* has *T. acaulis*-type characterized by a roughly striate or striato-rugulate surface, granulate colpus membranes, and generally larger grain size, while pollen grains of sect. *Trollius* has *T. europaeus*-type characterized by a finely striate surface, shortly echinate colpus membrane, and generally smaller grains. Our phylogenetic analysis also indicated that two sections have undergone different evolutionary routes. Furthermore, the sect. *Trollius* was divided into two groups according to the shape of the leaves. Leaves was palmately sect in one group and was palmately parted in the other group (Wang, 1979). There were actually two different evolutionary clades involved in sect. *Trollius* (clade II & clade III) in our phylogenetic analysis. However, taxa included in the clade II were leave-palmately-sect excluding *T. dschungaricus*, and taxa included in the clade III were leave-palmately-parted excluding *T. ranunculoides*, *T. vaginatus* and *T. micranthus*. In addition, Doroszewska (1974) defined seven sections within *Trollius*, of which sect. *Pumilotrollius*, *Longipetala*, *Trollius* were not monophyletic (**Figure 7**) in this study. It indicated that the genus *Trollius* were overly fine delineated based on floral morphology and distribution areas. Therefore, it is necessary to propose a more acceptable and practical classification system including more samples based on our phylogenetic results.

## 5 Conclusion

In this study, we successfully sequenced and assembled the complete plastomes of 16 *Trollius* species, providing valuable genomic resources for this genus. The plastomes of *Trollius* species were highly similar in terms of genome size, GC content, gene number, and gene types. Additionally, we observed very similar patterns of SC/IR boundaries in *Trollius* without a clear signal of expansion or contraction in our study. Using the whole plastomes, we constructed the phylogenetic relationships within the genus *Trollius.* The results showed that *Trollius* was a monophyletic group with three strongly-supported clade. Clade I was *T. lilacinus*, which was supported by many unique morphological characters, clade II and clade III were very consistent with their distribution areas. Besides, we identified 12 highly variable regions by comparative analysis of plastomes of *Trollius*, which may be used as specific DNA barcodes. Our findings lay a foundation for future research on phylogenetics, species identification, and conservation biology of *Trollius*. However, further investigation and research are needed to better understand the evolutionary history of this genus.

## Data availability statement

The datasets presented in this study can be found in online repositories. The names of the repository/repositories and accession number(s) can be found below: https://www.ncbi.nlm.nih.gov/genbank/, OR449279 https://www.ncbi.nlm.nih.gov/genbank/, OR449280 https://www.ncbi.nlm.nih.gov/genbank/, OR449281 https://www.ncbi.nlm.nih.gov/genbank/, OR449282 https://www.ncbi.nlm.nih.gov/genbank/, OR449283 https://www.ncbi.nlm.nih.gov/genbank/, OR449284 https://www.ncbi.nlm.nih.gov/genbank/, OR449285 https://www.ncbi.nlm.nih.gov/genbank/, OR449286 https://www.ncbi.nlm.nih.gov/genbank/, OR449287 https://www.ncbi.nlm.nih.gov/genbank/, OR449288 https://www.ncbi.nlm.nih.gov/genbank/, OR449289 https://www.ncbi.nlm.nih.gov/genbank/, OR449290 https://www.ncbi.nlm.nih.gov/genbank/, OR449291 https://www.ncbi.nlm.nih.gov/genbank/, OR449292 https://www.ncbi.nlm.nih.gov/genbank/, OR449293 https://www.ncbi.nlm.nih.gov/genbank/, OR449294.

## Author contributions

JL: Data curation, Writing – original draft, Conceptualization. YD: Conceptualization, Data curation, Writing – original draft. LX: Resources, Writing – original draft. XJ: Resources, Writing – original draft. ZZ: Data curation, Writing – original draft. MY: Conceptualization, Supervision, Writing – review & editing.
