# Supplementary material for: Comparative plastome genomics and phylogenetic relationships of the genus Trollius
Source: Front Plant Sci. 2023 Nov 17;14:1293091. doi: 10.3389/fpls.2023.1293091 (PMC10690957; doi:10.3389/fpls.2023.1293091)
Supplement: Supplementary file 1 [file DataSheet_1.docx]

Supplementary Material

Comparative Plastome Genomics and Phylogenetic Relationships of the genus *Trollius*

Jiaxin Li ^1†^, Yan Du ^1†^, Lei Xie ^2^, Xiaohua Jin ^3^, Zhirong Zhang ^4^, Meiqing Yang ^1*^

^1^ School of Pharmacy, Baotou Medical College, Baotou, Inner Mongolia, China

^2^ School of Ecology and nature conservation, Beijing forestry University, Beijing, China

^3^ State Key Laboratory of Systematic and Evolutionary Botany, Institute of Botany, Chinese Academy of Sciences, Beijing, China

^4^ Germplasm Bank of Wild Species, Kunming Institute of Botany, Chinese Academy of Sciences, Kunming, Yunnan, China

*** Correspondence:**Meiqing Yang
ymq40021011@163.com

**†**These authors have contributed equally to this work

# Supplementary Figures and Tables

## Supplementary Figures

**
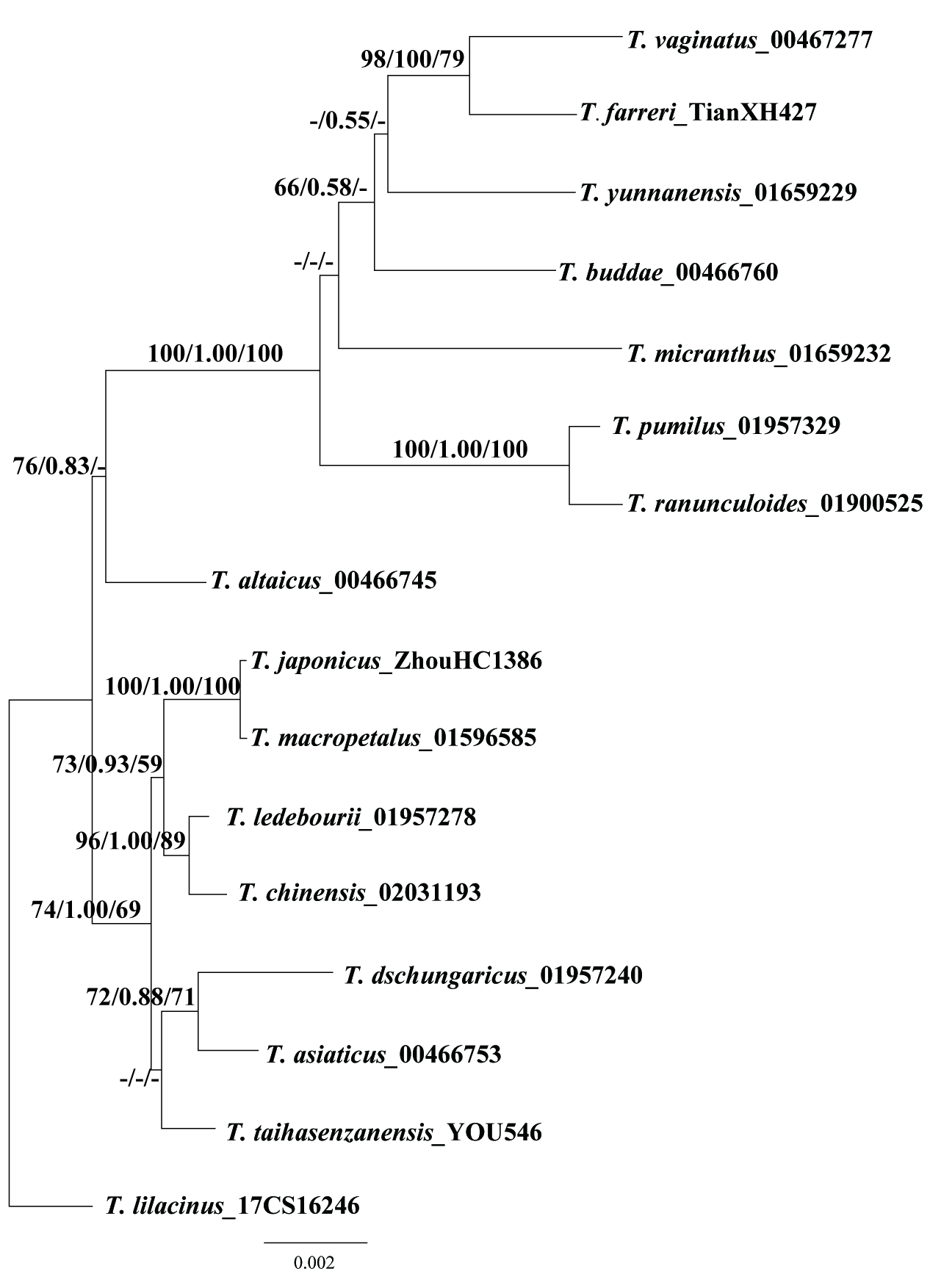
**

**Supplementary Figure 1.** The phylogenetic tree of the genus *Trollius* using maximum likelihood (ML), Bayesian inference and maximum parsimony (MP) methods based on nuclear ribosomal DNA (nrDNA).


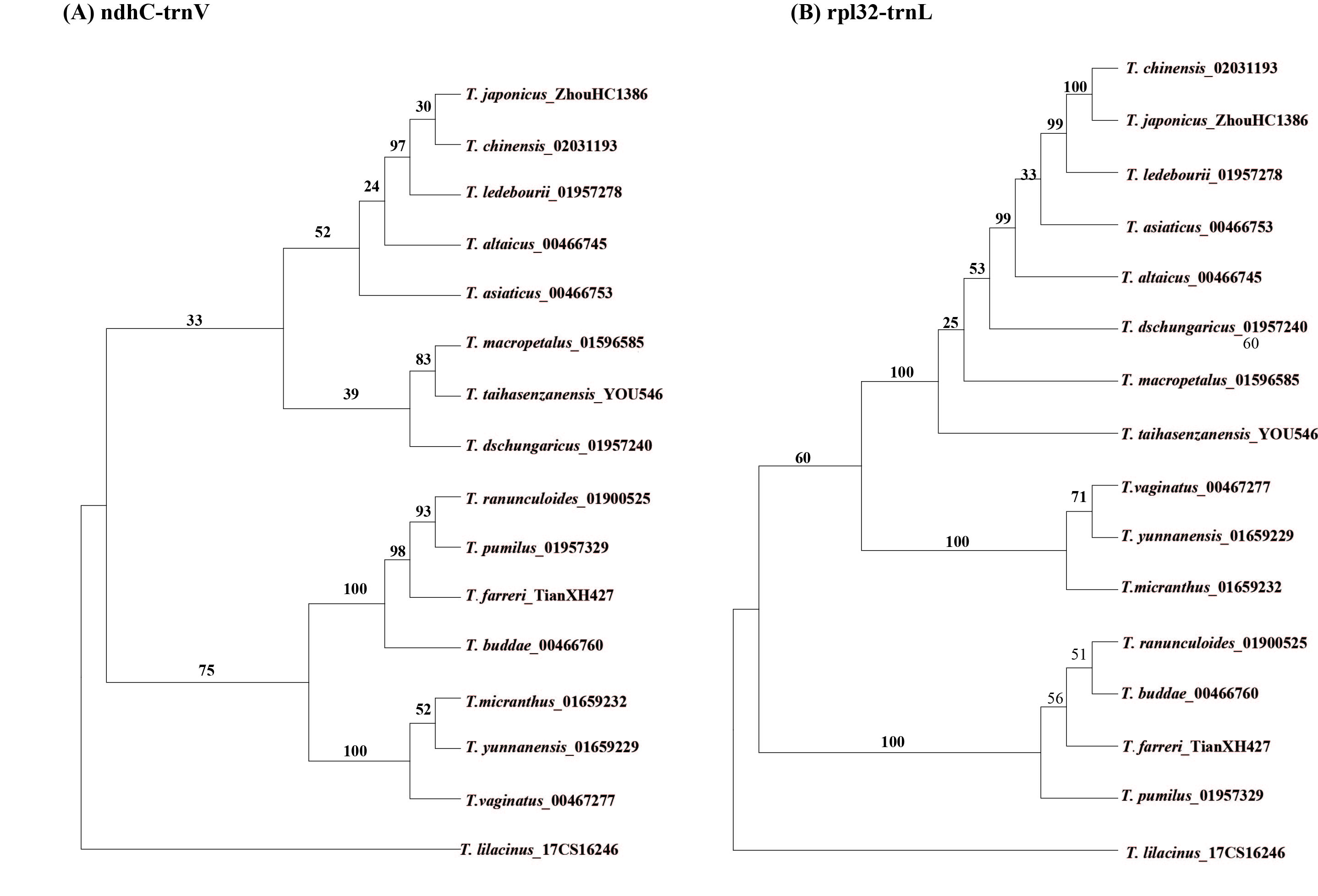

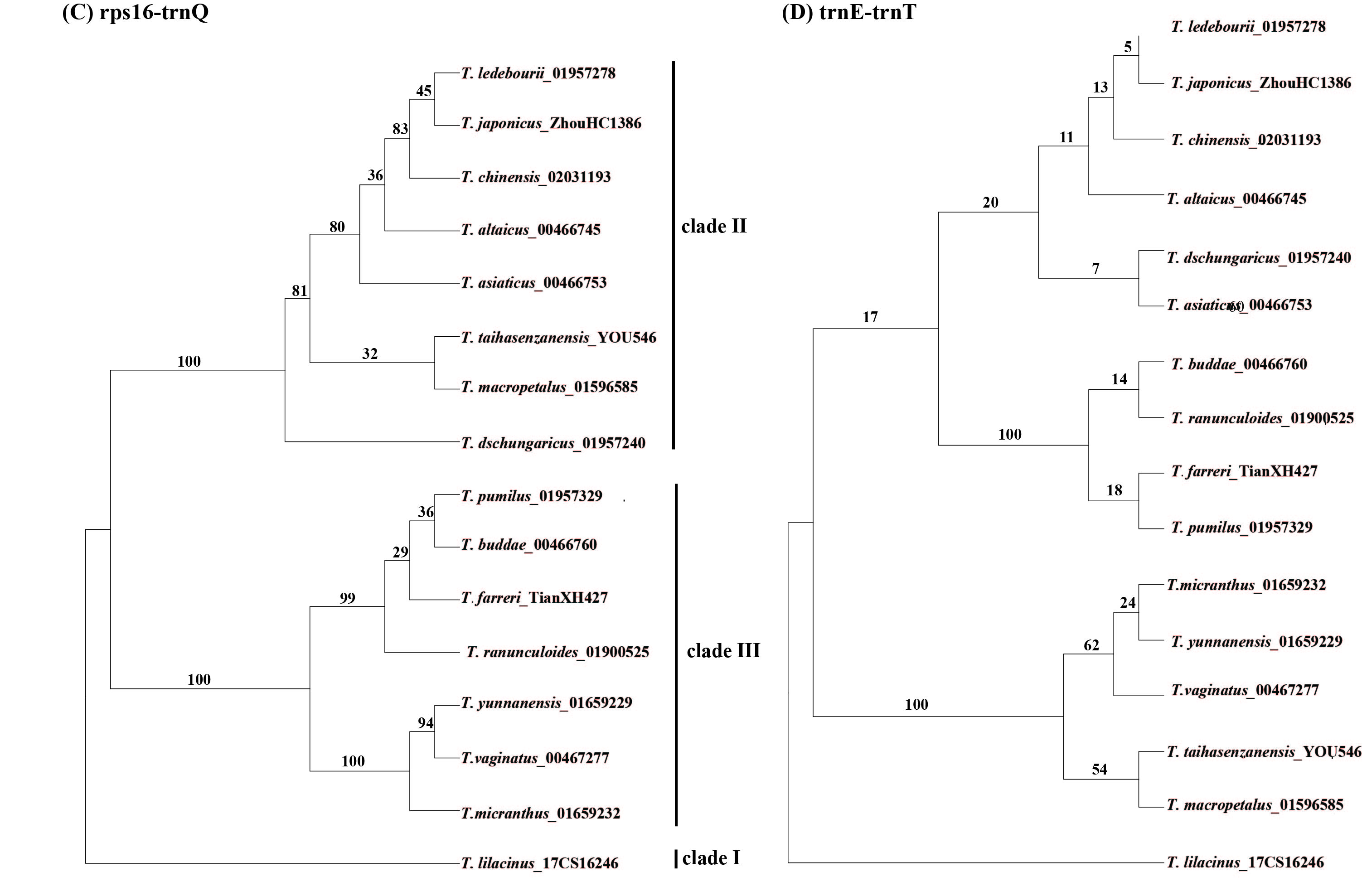

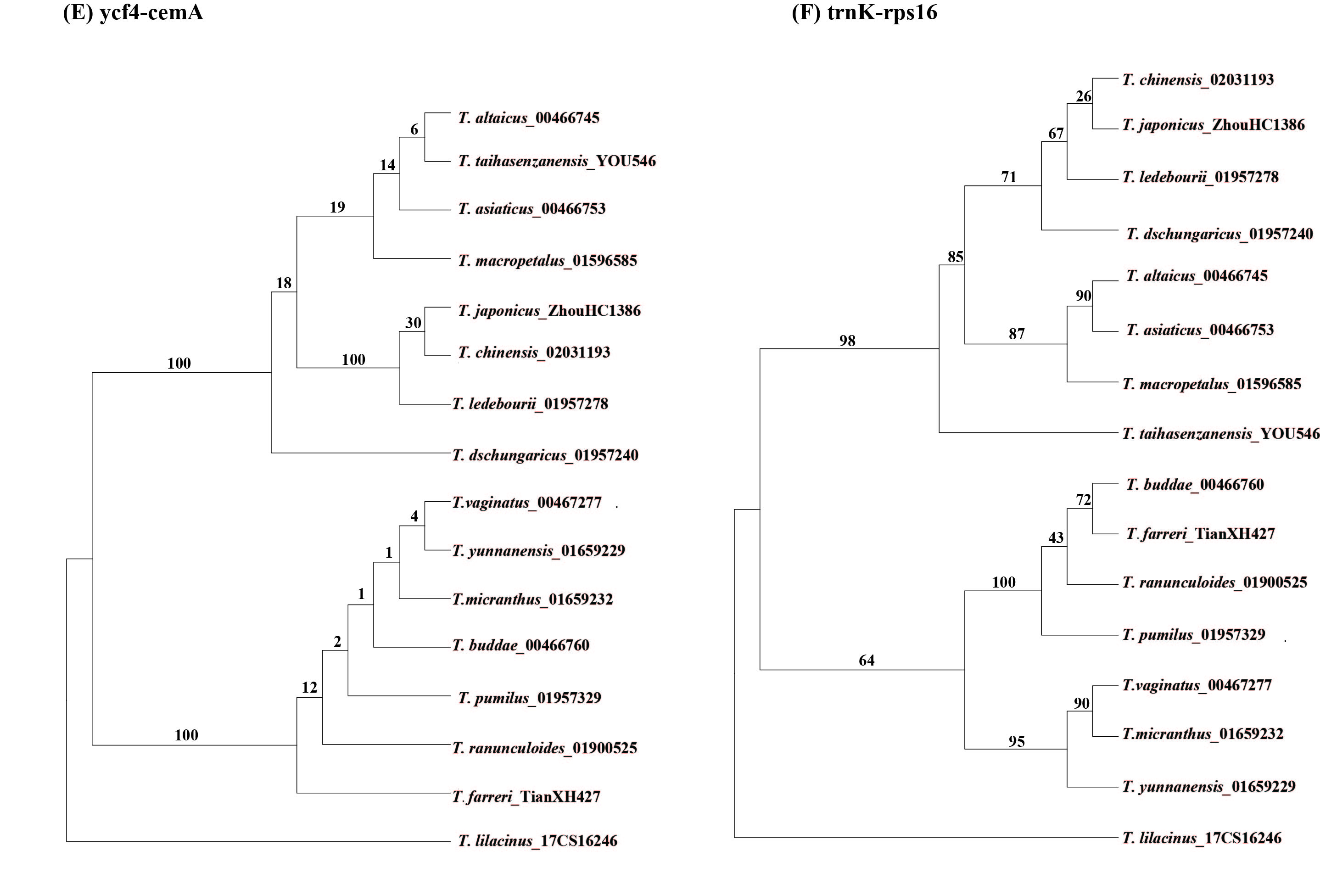

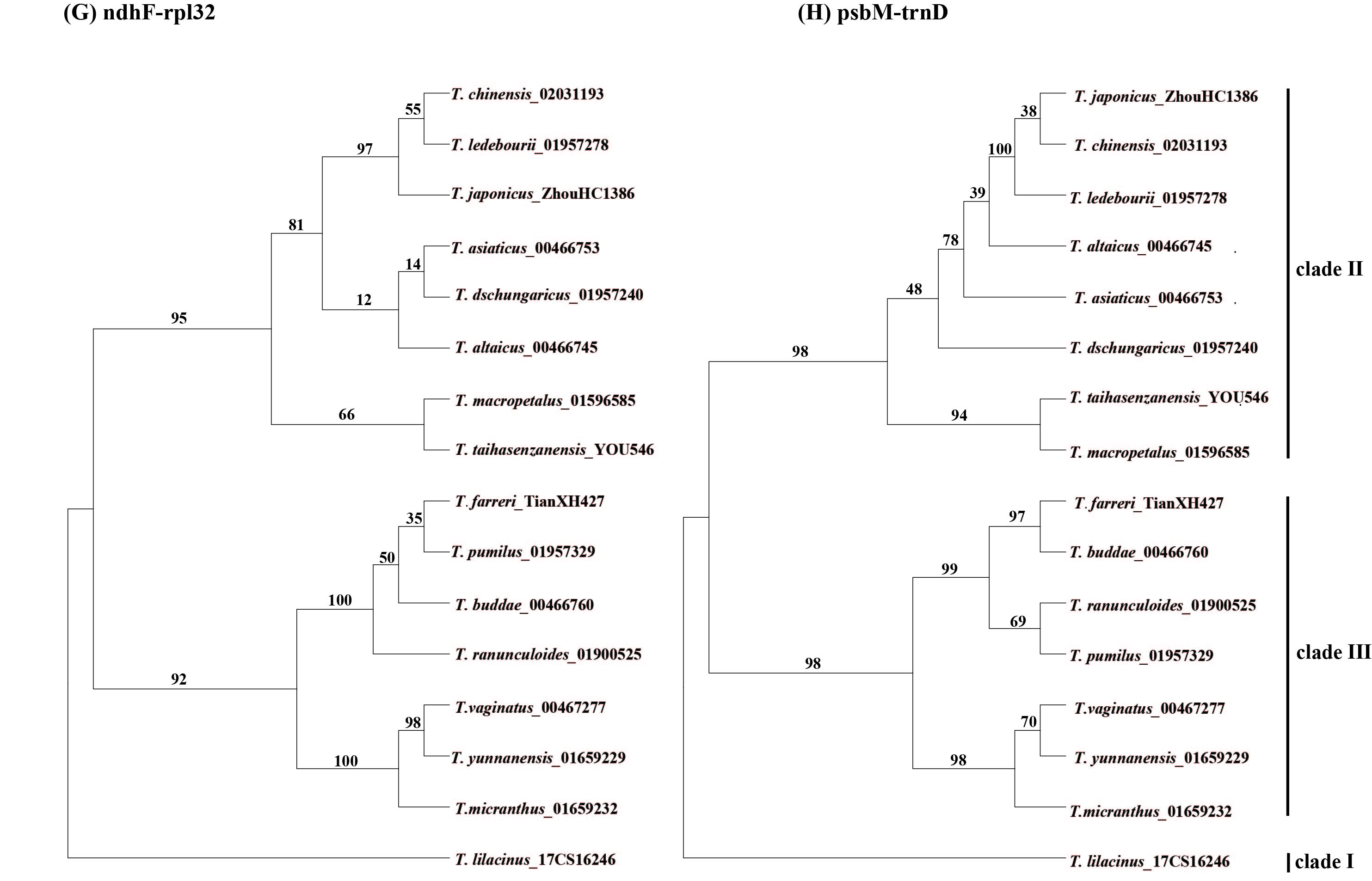

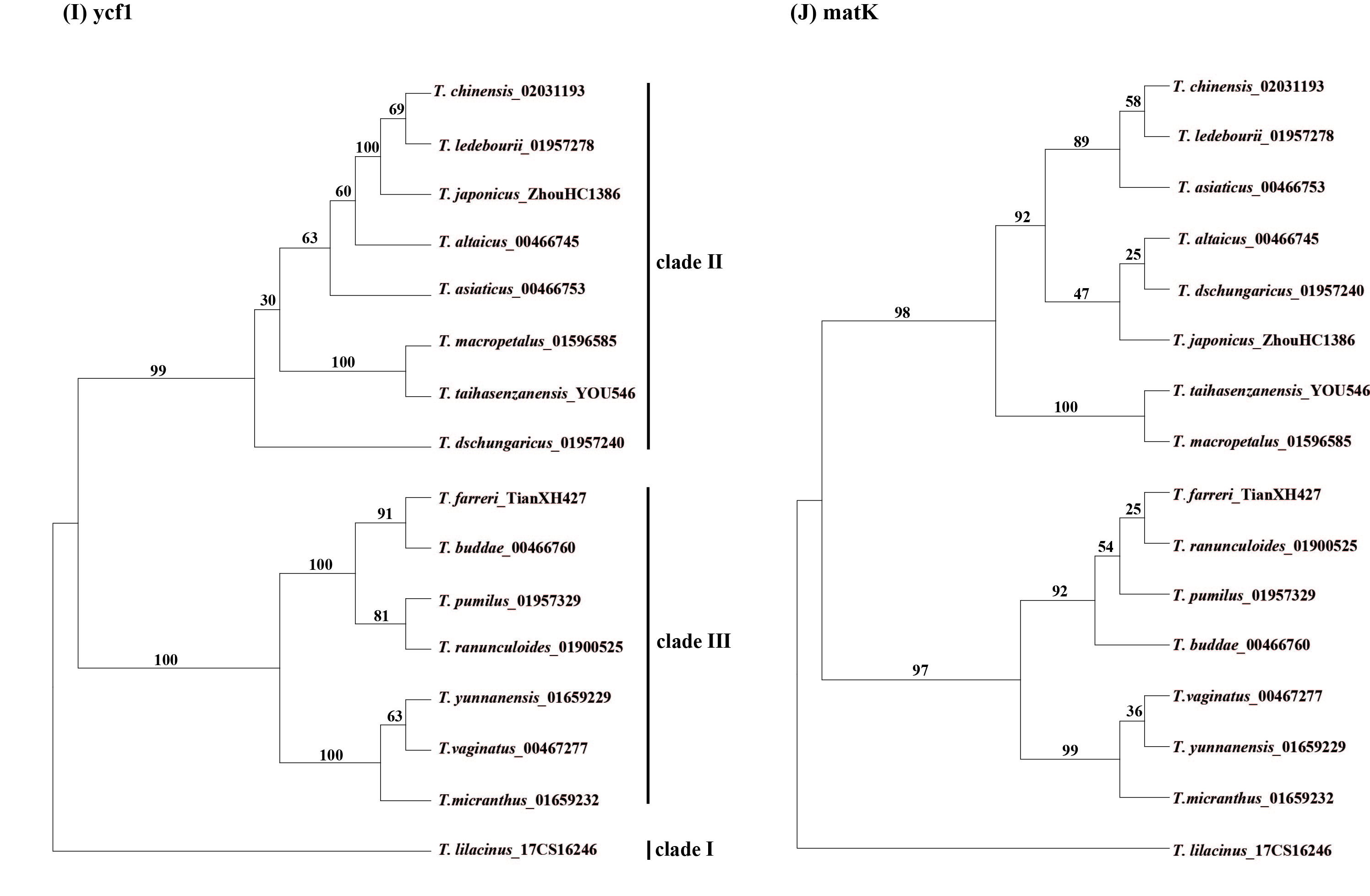

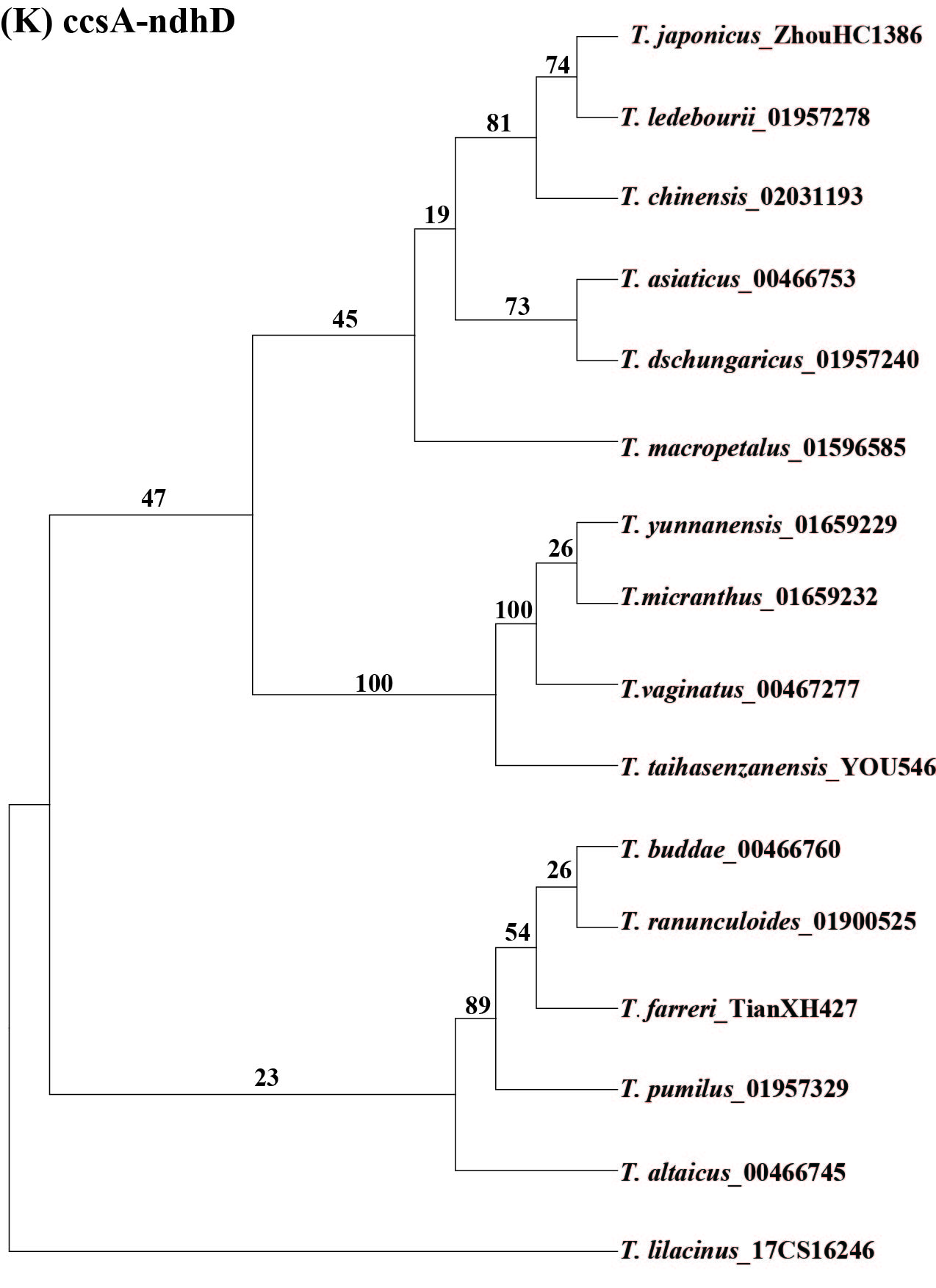


**Supplementary Figure 2.** The phylogenetic tree of the genus *Trollius* using maximum likelihood (ML) based on 11 highly polymorphic loci. **(A)** *ndhC*-*trnV*, **(B)** *rpl32*-*trnL*, **(C)** *rps16*-*trnQ*, **(D)** *trnE*-*trnT*, **(E)** *ycf4*-*cemA*, **(F)** *trnK*-*rps16*, **(G)** *ndhF*-*rpl32*, **(H)** *psbM*-*trnD*, **(I)** *ycf1*, **(J)** *matK*, **(K)** *ccsA*-*ndhD*.
